# Repolarization Changes Induced by Air Pollution in Ischemic Heart Disease Patients

**DOI:** 10.1289/ehp.7579

**Published:** 2005-01-14

**Authors:** Alexandra Henneberger, Wojciech Zareba, Angela Ibald-Mulli, Regina Rückerl, Josef Cyrys, Jean-Phillippe Couderc, Betty Mykins, Gabriele Woelke, H.-Erich Wichmann, Annette Peters

**Affiliations:** ^1^Ludwig-Maximilians-University of Munich, Munich, Germany; ^2^Institute of Epidemiology, GSF-National Research Center for Environment and Health, Neuherberg, Germany; ^3^Cardiology Unit, Department of Medicine, University of Rochester, Rochester, New York, USA; ^4^Focus-Network on Aerosols and Health, GSF-National Research Center for Environment and Health, Neuherberg, Germany

**Keywords:** air pollution, coronary disease, electrocardiography, epidemiology, repolarization

## Abstract

Epidemiologic studies report associations between particulate air pollution and cardiovascular morbidity and mortality, but the underlying pathophysiologic mechanisms are still unclear. We tested the hypothesis that patients with preexisting coronary heart disease experience changes in the repolarization parameters in association with rising concentrations of air pollution. A prospective panel study was conducted in Erfurt, East Germany, with 12 repeated electrocardiogram (ECG) recordings in 56 males with ischemic heart disease. Hourly particulate and gaseous air pollution and meteorologic data were acquired. The following ECG parameters reflecting myocardial substrate and vulnerability were measured: QT duration, T-wave amplitude, T-wave complexity, and variability of T-wave complexity. Fixed effect regression analysis was used adjusting for subject, trend, weekday, and meteorology. The analysis showed a significant increase in QT duration in response to exposure to organic carbon; a significant decrease in T-wave amplitude with exposure to ultrafine, accumulation mode, and PM_2.5_ particles (particles < 2.5 μm in aerodynamic diameter); and a corresponding significant increase of T-wave complexity in association with PM_2.5_ particles for the 24 hr before ECG recordings. Variability of T-wave complexity showed a significant increase with organic and elemental carbon in the same time interval. This study provides evidence suggesting an immediate effect of air pollution on repolarization duration, morphology, and variability representing myocardial substrate and vulnerability, key factors in the mechanisms of cardiac death.

Evidence from epidemiologic studies indicates that ambient particulate air pollution and air pollution episodes lead to increased cardiovascular hospital admissions and mortality (Pope 2000; Schwartz 1994). Although the relative effects of particulate air pollution are greater for respiratory than for cardiovascular mortality, the absolute number of deaths attributable to particulate air pollution is much higher for cardiovascular than for respiratory deaths (Dockery 2001; Frampton 2001).

Cardiac rhythm disorders are the leading cause of hospital admissions for cardiovascular diseases in the United States. More than half of the deaths due to ischemia, myocardial infarction, and cardiomyopathies are directly related to cardiac arrhythmias, and these deaths are usually sudden (Spooner et al. 2001; Zipes and Wellens 1998). Myocardial substrate (myocardial damage due to coronary disease, infarction, or cardiomyopathy), the autonomic nervous system (sympathetic activation or/and parasympathetic withdrawal), and myocardial vulnerability (ventricular arrhythmias, repolarization dynamics) are believed to be key factors that contribute to the mechanism of arrhythmogenic conditions and arrhythmic death (Zareba et al. 2001) and represent the so-called “cardiac death triangle.”

Repolarization abnormalities play a critical role in arrhythmogenesis, and electrocardiogram (ECG) measures of repolarization morphology and dynamics can identify patients at risk for cardiac death and sudden death (Atiga et al. 1998; Berger et al. 1997; Chevalier et al. 2003; Okin et al. 2000; Zabel et al. 1998). Previous studies on the daily variation of particulate air pollution and heart rate variability in elderly subjects showed an increase in heart rate (Liao et al. 2004; Peters et al. 1999; Pope et al. 1999) and a decrease in heart rate variability (Creason et al. 2001; Gold et al. 2000; Liao et al. 1999, 2004; Pope et al. 1999, 2004) associated with particulate air pollution. However, there are no clinical data regarding the influence of air pollution on repolarization parameters.

The purpose of this study was to assess associations between daily variations in particulate air pollution and repolarization ECG parameters representing abnormalities in the myocardial substrate and increased vulnerability of myocardium to arrhythmias. Novel measures of repolarization (e.g., T-wave complexity) were used to investigate the underlying pathophysiologic mechanisms leading to adverse reactions of the heart in response to air pollution.

## Materials and Methods

### Study population.

A prospective panel study was conducted in patients with coronary artery disease who participated in 12 subsequent clinical exams between 16 October 2000 and 27 April 2001 in Erfurt, a city of 200,000 inhabitants in East Germany. Subjects were required to be male, at least 50 years of age, and have doctor-diagnosed coronary artery disease confirmed by a typical history of angina pectoris or by prior myocardial infarction. Exclusion criteria were as follows: current smokers; patients with pacemakers; patients with recent (< 3 months) myocardial infarction, recent bypass surgery or coronary angioplasty, bundle-branch blocks, or type 1 diabetes; and patients on anticoagulation therapy (warfarin derivatives). Sixty-one coronary artery disease patients were recruited from a large clinical practice of a local cardiologist to participate in the study, and 56 patients remained for the analyses. Three patients with intermittent bundle-branch blocks and one patient with sinus arrhythmia were excluded, and one patient did not participate.

Data on health status, pulmonary and cardiac symptoms, medication, and smoking history were obtained at baseline and during follow-up visits. All subjects signed a written consent, and the study protocol was approved by the German ethics commission, Bayerische Landesaerztekammer.

### ECG recordings and analysis.

The ECG was recorded with a 12-lead Mortara H12 digital Holter recorder (Mortara Instrument, Milwaukee, WI, USA) using a digital sampling rate of 180 samples/sec per channel and included a 6-min period of rest in supine position with spontaneous breathing. The clinical visits were scheduled on the same weekday (Monday–Friday) and time (0800–1700 hr) for each patient once every 2 weeks to minimize the influence of circadian variation of the parameters studied. All methods used in the study were conducted according to standard operating procedures (SOP; available from the authors upon request).

The ECG recordings were analyzed manually by one observer for the QT interval and automatically for T-wave complexity and amplitude using the research version of the H-Scribe 12-Lead Digital Holter System (Mortara Instrument) at the University of Rochester Medical Center. The QT interval duration corrected for heart rate (QTc) was measured manually in lead II and adjusted for heart rate with Bazett’s formula (Bednar et al. 2001). We chose lead II for QT measurements because in clinical conditions this lead is considered representative for the overall electrical forces of the heart. For T-wave amplitude, we used original ECG leads I, II, and V1–V6, and the median value from those eight original leads was taken for each cardiac cycle and averaged over a 5-min period. T-wave complexity was measured in each beat by principal component analysis based on all 12 leads and averaged over a 5-min period. T-wave complexity describes the global shape of the T-wave with the advantage of not having the need for T-wave end determination. The first component (eigenvector) accounts for most of the energy in repolarization in a normal T-wave, whereas the second indicates a relevant contribution to the inhomogeneous repolarization. The average ratio between the second and the first component expressed as percentages represents a measure of complexity and heterogeneity of repolarization and provides an estimate of the fatness of the T-wave loop relative to its amplitude (Okin et al. 2002).

Variability of T-wave complexity was measured using an SD of this parameter over the 5-min period (similar to SD of normal-to-normal intervals for heart rate variability) and was not corrected for heart rate. However we also performed the analysis of the variability of T-wave complexity adjusted for heart rate variability (including the SD of normal-to-normal intervals as a linear term in the confounder model) similar to the approach for T-wave lability by Berger et al. (1997) and the work of Haigney et al. (2004) to test if the effect estimates of the variability of T-wave complexity were dominated or induced by the response of heart rate variability to air pollution.

### Air pollution monitoring.

The concentrations of ambient air pollutants were measured at a fixed monitoring site representing urban background levels according to the SOP developed within the framework of previous studies (Wichmann et al. 2000). Continuous ultrafine particle (UFP; 0.01–0.1 μm) counts, accumulation mode particle (ACP; 0.1–1.0 μm) counts, and PM_2.5_ (mass of particles < 2.5 μm in aerodynamic diameter) were measured with a mobile aerosol spectrometer consisting of the differential mobility analyzer combined with a condensation particle counter for particles with an aerodynamic diameter between 0.01 μm and 0.5 μm and an optical laser aerosol spectrometer for particles with a size range of 0.1–2.5 μm. Elemental carbon (EC) and organic carbon (OC) were determined hourly from an ambient carbon monitor (ACM 5400, Rupprecht & Patashnick, Co., Inc., Albany, NY, USA) after 17 December 2000.

Continuous data on meteorologic variables of temperature, barometric pressure, and relative humidity as well as sulfur dioxide, nitrogen dioxide, nitrogen monoxide, and carbon monoxide were collected from existing networks. Missing values in the ambient particle pollutants UFPs and PM_2.5_ between 20 January and 13 February 2001 and one missing day (31 March 2001) in the meteorologic variables of temperature and relative humidity were either imputed by a linear regression model (UFPs) or replaced based on corrected parallel measurements with other devices (PM_2.5_, temperature, relative humidity). The squared multiple correlation for the UFP regression models was 0.96.

### Statistical analyses.

The study was conducted as a panel study with repeated measurements, a longitudinal study in which the cohort was followed for a certain time, and the same persons were measured repeatedly (12 times). Thus, every person acts as his own control, which automatically provides an adjustment for intraindividual variability. Panel studies are most effective for studying short-term health effects of air pollution, particularly in susceptible subpopulations.

Data were analyzed using SAS statistical package (version 8.2; SAS Institute Inc., Cary, NC, USA) and S-Plus (version 6.0; MathSoft Inc., Cambridge, MA, USA). We used generalized additive models (GAMs), including pollutant and confounder variables, for fixed effects regression with individual intercepts for each patient. GAM was used to adjust for confounders with nonparametric smooth functions based on locally weighted least squares. Model building was done for each ECG variable separately. Model fit was based on the Akaike Information Criterion (AIC). The best nonparametric model was compared with parametric modeling (linear, polynomials of second or third order) for each confounder variable. We considered the following confounder variables: an indicator variable for each subject, long-term time trend, temperature, relative humidity, barometric pressure, and weekday of the visit. Sensitivity analyses performed were either by changing the confounder model or by excluding specific patients.

The exposure, in this case ambient particulate and gaseous air pollution, was measured in different time intervals before the ECG measurements because current knowledge on the underlying biologic mechanisms supports an immediate as well as a cumulative response over 5 days. For every patient, we analyzed the individual 0–5, 6–11, 12–17, 18–23, and 0–23 hr and 2–5 days of air pollution exposure before the ECG recording to look for immediate and delayed responses of the patients. No adjustment was made for autocorrelation in the patients’ data or in the pollution data because the repeated measurements took place 2 weeks apart. The model with the lowest AIC was selected for each outcome variable. Effect estimates are presented together with 95% confidence intervals (CIs) based on an increase in air pollution concentration from the first to the third quartile [interquartile range (IQR)].

## Results

### Patient characteristics and repolarization parameters.

The study group consisted of 56 male patients with a mean (± SD) age of 66 ± 6 years (range, 52–76 years) and a mean (± SD) body mass index (BMI) of 28 ± 4 kg/m^2^ (range, 22–38 kg/m^2^). Most patients had prior myocardial infarction, and all patients had stable coronary artery disease. The patients were current nonsmokers, but almost three-fourth were ex-smokers. They were treated mainly with β-blockers or angiotensin-converting enzyme (ACE) inhibitors (Table 1). Medication was maintained without changes during the study except for 10 patients (18%) who had an update in medication (either removed or added prescription).

Subjects had clinical exams every 2 weeks for 6 months, and 662 (99%) of the targeted 672 clinical exams were conducted; 625 (93%) ECG recordings were available for analysis. Table 1 shows the average values of the studied ECG parameters. QTc was not significantly correlated (Spearman correlation coefficient *r*) with the other three ECG parameters. The highest correlation could be seen between the T-wave complexity and its variability (*r* = 0.58). T-wave amplitude showed a negative correlation with T-wave complexity and with the variability of T-wave complexity (*r* = −0.42 and *r* = −0.49, respectively).

### Air pollutants.

**Figure 1:**
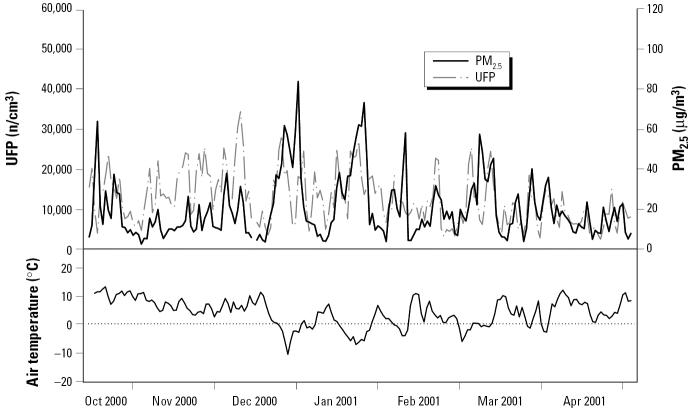
Time series of UFP and PM_2.5_ concentrations together with air temperature.

A description of particulate and gaseous air pollutants, as well as meteorologic variables, is shown in Table 2. Figure 1 shows UFP and PM_2.5_ concentrations and air temperature during the study period. Although not strongly correlated, UFPs were more correlated (Spearman correlation coefficient *r*) with ACPs (*r* = 0.64) than with PM_2.5_ (*r* = 0.41). ACPs and PM_2.5_ were highly correlated (*r* = 0.91). EC and OC were both highly correlated with ACPs and PM_2.5_ (*r* > 0.76). The correlation between gaseous and particulate air pollutants was highest for UFPs with NO (*r* = 0.83) and NO_2_ (*r* = 0.76); for ACPs with NO (*r* = 0.68), NO_2_ (*r* = 0.69), and CO (*r* = 0.82); for PM_2.5_ with CO (*r* = 0.72); and for OC and EC with NO, NO_2_, and CO (*r* > 0.68).

### Analysis of the association between air pollution and repolarization parameters.

Table 3 shows the estimated effects for particulate air pollution concentrations (UFPs, ACPs, PM_2.5_) in different time windows on the repolarization parameters together with the 95% CIs. In order to base values on comparable increments of exposure for the study period, the effect estimates were multiplied by the IQR of the pollutants (Table 2).

For all analyzed particulate concentrations measured during the 24 hr before the ECG recording, there was an increase of QTc, which was significant only for ACP concentrations during the 6–11 hr before the recordings and for OC for all the 6-hr time windows within the first 18 hr before the recording and for the complete 0–23 hr period.

T-wave amplitude showed a significant decrease in association with UFP, ACP, and PM_2.5_ concentrations during the 0–5 hr before the ECG, and UFPs as well as ACPs also showed significant effects for concentrations during the 0–23 hr before the recording. For PM_2.5_, this 24-hr average effect size was comparable but not significant. With EC concentrations an immediate (lag of 0–5 hr) borderline decrease of T-wave amplitude could be seen.

Correspondingly T-wave complexity showed a significant increase in association with PM_2.5_ concentrations during the 0–5 hr and with UFPs during the 18–23 hr before the recording.

The variability of T-wave complexity showed a borderline increase with PM_2.5_ concentrations during the 0–5-hr and the 0–23-hr periods before ECG measurement and a significant increase for OC and EC concentrations during 0–5 (only borderline for OC), 12–17, and 0–23 hr before the ECG recording.

**Figure 2:**
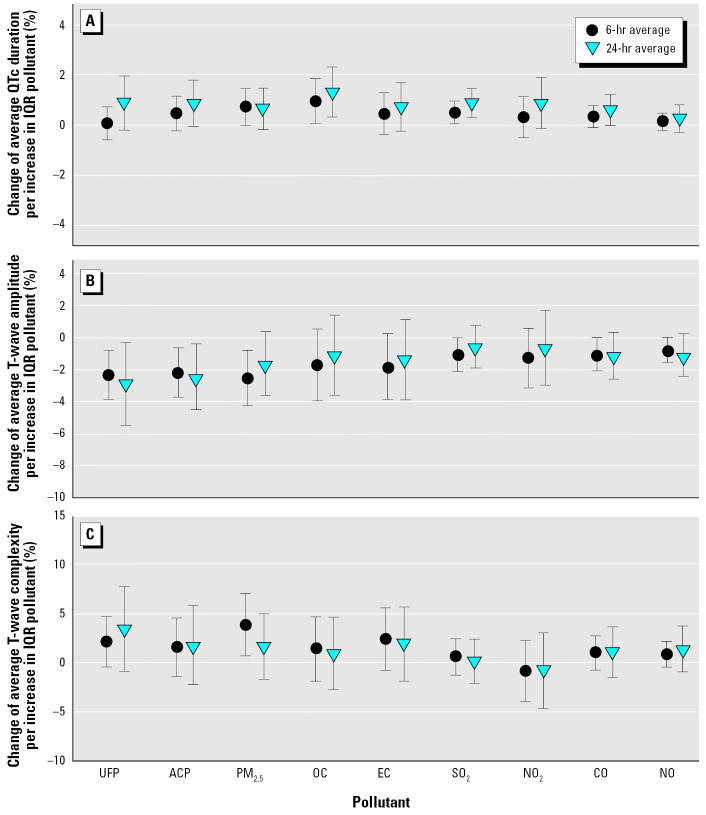
Effect estimates with 95% CIs of particulate and gaseous air pollution during the 6 hr and 24 hr before the recording of (*A*) QTc interval, (*B*) T-wave amplitude, and (*C*) T-wave complexity.

Generally, effects of gaseous pollutants were weaker. For QTc, an association with NO_2_ (effect estimate, 4.47; 95% CI, 1.02–7.93) and CO (3.86; 95% CI, 0.92–6.81) concentrations during 6–11 hr before the recording and with SO_2_ (3.75; 95% CI, 1.21–6.28) during the whole 0–23 hr before ECG were observed. NO, a gaseous marker for freshly emitted UFPs from diesel vehicles, showed an association between concentrations during 0–23 hr before the ECG and the variability of T-wave complexity (effect estimate, 0.14; 95% CI, 0.02–0.26) and between concentrations during 0–5 hr before the recording and the T-wave amplitude (−1.97; 95% CI, −3.91 to −0.03). No further significant associations between T-wave parameters and gaseous pollutants were found (Figure 2).

**Figure 3:**
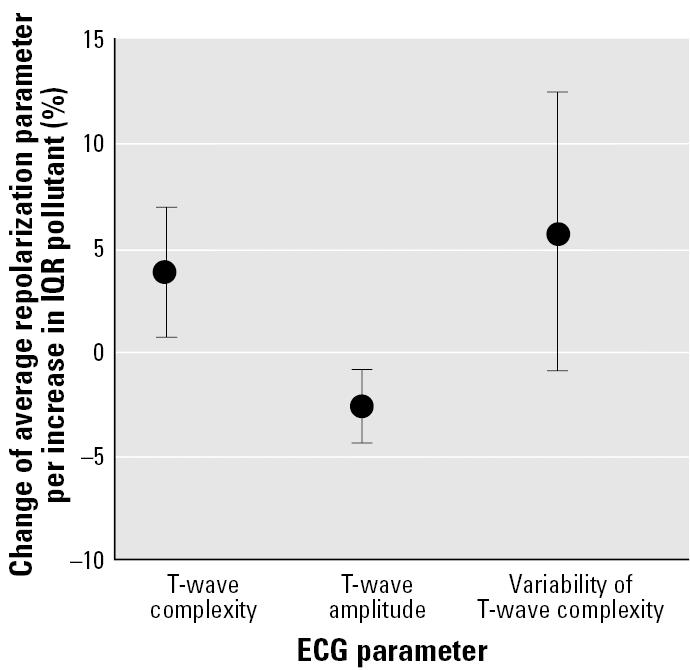
Effect estimates with 95% CIs of PM_2.5_ concentrations during the 6 hr before the recording on T-wave parameters reflecting myocardial substrate and vulnerability.

When comparing the influence of PM_2.5_ concentrations during the first 6 hr before the recording on T-wave parameters (scaled to the percent change of average repolarization parameter), T-wave complexity showed larger effects than did T-wave amplitude (Figure 3). Besides some isolated effects possibly due to multiple testing, no significant delayed effects could be seen for any ECG parameters for time intervals previous to the 24-hr lag.

A sensitivity analysis for the variability of T-wave complexity by including an additional adjustment for heart rate variability in the confounder model showed identical effect estimates. Even stronger effects in the same direction were observed when excluding from our analysis two patients taking antiarrhythmic medication that may affect repolarization. In a further sensitivity analysis, all results were consistent when leaving out either the trend or the meteorology in the confounder model. A sensitivity analysis with parametric smoothing functions (natural splines) instead of nonparametric smoothing functions (loess) for the confounder model did not show any changes in the effect estimates.

## Discussion

This longitudinal study showed that increased levels of particulate air pollution are associated with significant changes in ECG repolarization parameters reflecting myocardial substrate and vulnerability. The analyzed repolarization parameters showed different pollutant-specific responses, although some of the measured particulate pollutants were highly correlated. In association with ACP, PM_2.5_, and OC concentrations for the whole 0–23-hr period before the recording, an increase in QTc could be seen, which was significant especially for OC. T-wave amplitude showed a significant decrease with UFP, ACP, PM_2.5_, and EC concentrations measured during 0–5 hr (only borderline for EC) and during 0–23 hr before the recording (significant only for UFPs and ACPs). Consistently with this finding, T-wave complexity, a computerized measure of repolarization morphology, increased significantly in association with PM_2.5_ concentrations in the same time interval. The variability of T-wave complexity also showed an immediate borderline increase with PM_2.5_ concentrations measured during the 0–5 and the 0–23-hr time interval before the ECG and a significant increase in association with OC and EC concentrations during the whole 24 hr before recording the ECG. The observed inhomogeneous reaction to the air pollution mix for the analyzed repolarization parameters might point toward different actions of air pollution components on the complex repolarization process (Brook et al. 2004).

Previous studies on the daily variation of particulate air pollution and heart rate variability in elderly subjects showed an increase in heart rate (Liao et al. 2004; Peters et al. 1999; Pope et al. 1999) and a decrease in heart rate variability (Creason et al. 2001; Gold et al. 2000; Liao et al. 1999, 2004; Pope et al. 1999, 2004) associated with particulate air pollution. Animal data support the concept that the autonomic nervous system may be a target for the adverse effects of air pollution. Rats exposed to fuel oil fly ash, a known contributor to PM_2.5_, developed an inflammation in the lung and also showed signs of inflammatory response in the heart (Killingsworth et al. 1997). Instillation of residual oil fly ash caused cardiac arrhythmias in rats with preexisting pulmonary inflammation (Watkinson et al. 1998). T-wave alternans, a marker of myocardial vulnerability and electrical instability, increases in dogs with inhalation of residual oil fly ash (Godleski et al. 2000). Exposure to concentrated air particles induced an increase of the high- and low-frequency domains of the heart rate variability in healthy dogs (Godleski et al. 2000). All of these findings suggest that inhaled particles may affect the balance between the sympathetic and parasympathetic control of the heart, and promote a stress response that potentially leads to arrhythmias.

This study demonstrates that air pollution also affects the other two components of the “cardiac death triangle,” the myocardial substrate and myocardial vulnerability, assessed by repolarization parameters. Abnormalities in repolarization morphology, described by the fairly novel measurement of T-wave complexity and T-wave amplitude, reflect the status of the myocardial substrate and were previously found to be associated with an increased risk of cardiac events in a cohort of healthy subjects (Kors et al. 1998; Okin et al. 2002) and in postinfarction patients (Zabel et al. 1998, 2000) in studies unrelated to the field of air pollution. Our findings indicate that abnormalities in repolarization duration and morphology occur in response to an increased level of air pollution reflecting its short-term influence on the myocardium. Such changes might predispose to arrhythmic events. The risk of an arrhythmic event is further enhanced by the observed increased vulnerability of the myocardium (measured by the variability of T-wave complexity). Further evidence for this interpretation was collected by a study in patients with implanted cardioverter defibrillators, where increases in levels of ambient particles have been associated with increases in arrhythmia frequency (Peters et al. 2000). Also, Riediker et al. (2004) found a strong increase of ventricular and supraventricular ectopic beats with higher PM_2.5_ in-vehicle measurements in young, healthy, nonsmoking male highway patrol troopers.

In the study presented here, most of the changes in repolarization due to elevated air pollution (UFPs, ACPs, PM_2.5_, OC, EC) started immediately. A decrease in heart rate variability with an increase in PM_2.5_ was analyzed in Pope et al. (1999) for the same day and the previous day. Gold et al. (2000) also showed immediate effects for PM_2.5_ concentrations during 0–3 hr before the testing, which is consistent with our findings 0–5 hr before the recording. Peters et al. (2000) found an association between cardiac arrhythmia and a 2-day lag for PM_2.5_, CO, and NO_2_. Pekkanen et al. (2002) showed an association between ACPs and ST-segment depression in the ECG on the 2-day lag. These findings lead to the hypothesis that the immediate effects in T-wave morphology and heart rate variability might lead 2 days later to a manifestation expressing in ST-segment depression or cardiac arrhythmia.

Particulate matter is a complex mixture containing many different components including metallic compounds such as iron, zinc, copper, vanadium, and nickel, which are potent inducers of physiologic effects in animals and humans (Campen et al. 2002; Evangelou and Kalfakakou 1993). Direct effects on the cardiovascular system, blood, and lung receptors, as well as indirect effects mediated through pulmonary oxidative stress and inflammatory responses, have been observed. The direct effects may occur via agents such as soluble constituents of PM_2.5_ or possibly UFPs that cross the pulmonary epithelium into the circulation. They might be an explanation for the quick cardiovascular responses within a few hours, as seen, for example, for the repolarization and T-wave morphometry changes in this study. Mechanisms under discussion (Brook et al. 2004; Schulz et al., in press) include a dysfunction of the autonomic nervous system in response to direct reflexes from receptors in the lung, a cardiac malfunction due to ischemic responses in the myocardium, and/or altered ion channel functions in the myocardial cells. Complicating our understanding even more is the fact that different chemicals released from deposited particles are able to induce different ion channel reactions (Graff et al. 2004). The observed change in QT interval and T-wave morphology might be indicative of changes in ion channel functions, but the understanding of the complex biologic pathways is still very limited. In contrast, responses within several hours or days after the inhalation of particles may possibly occur via pulmonary oxidative stress and systemic inflammation. These reactions are able to trigger endothelial dysfunction and a procoagulatory state with thrombus formation and promotion of atherosclerotic lesions.

It is conceivable that the combined effect of transient increases in blood coagulability (Seaton et al. 1995), an acute-phase response (Ernst and Resch 1993; Ridker et al. 2000), an increase in blood viscosity (Peters et al. 1997), and a change in myocardial substrate and vulnerability, as suggested in this study, could precipitate adverse cardiac events, especially in susceptible individuals.

### Strength and limitations.

Our longitudinal study with multiple observations per subject has the advantage of an almost complete follow-up in all 56 participants for 6 months, with each subject being his own control. To assure the quality of the ECGs, all nurses were trained carefully and frequently checked by the quality assurance officer in terms of electrode placement as well as skin preparation. Because the ECG parameters differ from person to person (age, sex, genetics, physiology, pathophysiology, therapy), the confounder model included an indicator variable for each subject and thus indirectly adjusted for the interperson differences and medication use of the participants. Ischemic heart disease patients are of particular interest because their cardiovascular disease might make them particularly sensitive to the effects of air pollution episodes, and these patients are particularly prone to die from cardiac and arrhythmic death. The often high use of different medications in these patients is expected to bias the estimates toward the null and thus only leads to an underestimation of the effects. For subgroup analyses of different medication groups, the sample size and thus the statistical power were unfortunately too small.

We chose to focus on T-wave complexity and amplitude in addition to the QTc interval to evaluate changes in repolarization morphology and duration as more reliable descriptors of repolarization changes than QT duration. Accumulating evidence indicates that principal component analysis of the T-wave provides a better quantitative assessment of the complexity of repolarization than other ECG parameters and is becoming a useful tool in noninvasive ECG diagnostics (Okin et al. 2002). The medical relevance of the observed changes in the analyzed ECG parameters might be small, but the results of this study will nevertheless help to understand the different pathways and mechanisms of the “cardiac death triangle” that lead from ambient air pollution to adverse cardiac events.

Misclassification of air pollution exposure is another potential source of bias especially in time-series studies (Zeger et al. 2000). Factors such as wind direction, climatic conditions, long-range transport, and distances from sources affect personal exposure patterns to pollutants from ambient sources. But any exposure misclassification would be expected to be nondifferential and thus to bias the estimates toward the null.

## Conclusion

Elevated particulate air pollution has a significant immediate effect on QTc duration, T-wave complexity, and T-wave amplitude. There is a convincing body of evidence that particulate air pollution is associated with an increased risk of cardiovascular morbidity and mortality, and this study provides evidence for a deleterious effect of air pollution on myocardial substrate and vulnerability, key factors in the mechanisms of cardiac death.

## Figures and Tables

**Table 1:** Description of the study population (56 male subjects with a history of coronary artery disease).

Clinical and ECG characteristics	Mean ± SD or total (%)
Clinical characteristics	
Age (years)	66 ± 6
BMI (kg/m^2^)	28 ± 4
Past myocardial infarction	43 (77)
Type 2 diabetes mellitus	12 (21)
Revascularization (CABG/PTCA)	48 (86)
COPD	5 (9)
Hypertension	39 (70)
NYHA ≥II	17 (30)
Occupational status	
Work part time or full time	4 (7)
Retired	49 (88)
Unable to work or unemployed	3 (5)
Exposed to toxic gases, dust, or fumes during work	1 (2)
Smoking	
Never smoker	15 (27)
Ex-smoker	41 (73)
Medication use	
β-Blockers	43 (77)
ACE inhibitors	30 (54)
Calcium blockers	17 (30)
Nitrate	24 (43)
Statins and fibrates	29 (52)
ECG parameters	
Heart rate (beats/min)	64.7 ± 9.9
QTc interval*^a^* (msec)	428.3 ± 35.6
T-wave complexity (%)	21.6 ± 12.8
Variability of T-wave complexity (%)	2.5 ± 1.8
T-wave amplitude*^b^* (μV)	256.0 ± 126.9

Abbreviations: CABG/PTCA, coronary artery bypass graft surgery/percutaneous transluminal coronary angioplasty (both procedures had to be performed more than 3 months before enrollment); COPD, chronic obstructive pulmonary disease; NYHA, New York Heart Association classification.

a Manually measured from lead II, Bazett corrected.

b Median value from leads I, II, and V1–V6.

**Table 2:** Daily concentration of air pollutants and meteorologic variables between 12 October 2000 and 27 April 2001.

Variable	No.	Mean ± SD	Minimum	25%	Median	75%	Maximum	IQR
UFP (n/cm^3^)*^a^*	196	12,602 ± 6,455	2,542	7,326	11,444	17,332	34,294	10,005
ACP (n/cm^3^)	167	1,593 ± 1,034	328	821	1,238	2,120	4,908	1,299
PM_2.5_ (μg/m^3^)*^b^*	197	20.0 ± 15.0	2.6	9.7	14.9	26.1	83.7	16.4
OC (μg/m^3^)*^c^*	126	1.5 ± 0.6	0.3	1.1	1.4	1.8	3.4	0.7
EC (μg/m^3^)*^c^*	126	2.6 ± 2.4	0.2	1.0	1.8	3.2	12.4	2.3
SO_2_ (μg/m^3^)	198	4.1 ± 1.8	3.0	3.0	3.4	4.6	11.7	1.5
NO_2_ (μg/m^3^)	198	34.3 ± 11.4	8.0	25.3	34.0	42.5	68.4	17.2
CO (mg/m^3^)	198	0.52 ± 0.29	0.11	0.33	0.44	0.60	1.93	0.27
NO (μg/m^3^)	196	24.3 ± 27.8	4.0	6.8	12.3	30.3	137.6	23.5
Temperature (°C)*^d^*	198	4.1 ± 4.8	−10.4	0.5	4.4	7.9	13.2	7.4
Barometric pressure (hPa)	198	973.4 ± 9.7	949.5	966.3	972.9	980.0	995.7	13.6
Relative humidity (%)*^d^*	198	83.5 ± 8.8	55.8	78.9	84.3	88.8	100.0	10.0

a 621 (13%) of the hourly measurements were imputed.

b 715 (15%) of the hourly measurements were imputed.

c Measurements started 18 December 2000.

d 24 (0.5%) of the hourly measurements were imputed.

**Table 3:** Effect estimates of particulate air pollution on repolarization parameters reflecting myocardial substrate and vulnerability per IQR (95% CI).

Hours before recording	QTc interval (msec)	T-wave amplitude (μV)	T-wave complexity (%)	Variability of T-wave complexity (%)
UFP				
0–5 hr	0.16 (^−^2.67–2.98)	^−^5.91 (^−^9.80–^−^2.01)^#^	0.46 (^−^0.09–1.01)^*^	0.06 (^−^0.07–0.20)
6–11 hr	3.59 (^−^0.69–7.87)	^−^5.90 (^−^12.01–0.21)^*^	0.41 (^−^0.44–1.26)	0.04 (^−^0.17–0.25)
12–17 hr	1.98 (^−^1.71–5.68)	^−^1.91 (^−^7.17–3.35)	0.01 (^−^0.71–0.73)	0.04 (^−^0.14–0.22)
18–23 hr	3.07 (^−^0.77–6.91)	^−^2.53 (^−^7.85–2.78)	0.76 (0.03–1.49)^**^	0.09 (^−^0.09–0.27)
0–23 hr	3.77 (^−^0.97–8.52)	^−^7.30 (^−^13.97–^−^0.63)^**^	0.74 (^−^0.19–1.66)	0.10 (^−^0.13–0.34)
ACP				
0–5 hr	1.87 (^−^1.13–4.87)	^−^5.54 (^−^9.51–^−^1.57)^#^	0.34 (^−^0.30–0.97)	0.12 (^−^0.04–0.27)
6–11 hr	5.15 (0.95–9.36)^**^	^−^5.34 (^−^10.93–0.26)^*^	^−^0.01 (^−^0.89–0.86)	0.14 (^−^0.07–0.36)
12–17 hr	3.36 (^−^0.32–7.03)^*^	^−^3.64 (^−^8.52–1.23)	0.17 (^−^0.58–0.92)	0.17 (^−^0.02–0.35)^*^
18–23 hr	2.12 (^−^1.41–5.67)	^−^3.95 (^−^8.67–0.76)	0.38 (^−^0.35–1.11)	0.12 (^−^0.05–0.30)
0–23 hr	3.70 (^−^0.35–7.75)^*^	^−^6.31 (^−^11.66–^−^0.95)^**^	0.38 (^−^0.49–1.25)	0.17 (^−^0.04–0.38)
PM_2.5_				
0–5 hr	3.06 (^−^0.23–6.35)^*^	^−^6.46 (^−^10.88–^−^2.04)^#^	0.84 (0.17–1.51)^**^	0.15 (^−^0.02–0.31)^*^
6–11 hr	2.83 (^−^0.80–6.45)	^−^2.00 (^−^6.95–2.96)	^−^0.04 (^−^0.77–0.98)	0.05 (^−^0.13–0.23)
12–17 hr	1.68 (^−^1.22–4.59)	^−^0.72 (^−^4.77–3.33)	^−^0.07 (^−^0.62–0.48)	0.07 (^−^0.05–0.18)
18–23 hr	1.11 (^−^1.95–4.16)	^−^3.99 (^−^8.22–0.24)^*^	0.25 (^−^0.32–0.83)	0.08 (^−^0.04–0.20)
0–23 hr	2.77 (^−^0.90–6.44)	^−^4.11 (^−^9.13–0.90)	0.35 (^−^0.37–1.07)	0.14 (^−^0.01–0.28)^*^
OC				
0–5 hr	4.15 (0.22–8.09)^**^	^−^4.31 (^−^10.07–1.44)	0.31 (^−^0.39–1.01)	0.12 (^−^0.01–0.26)^*^
6–11 hr	4.72 (0.20–9.24)^**^	0.50 (^−^6.30–7.30)	^−^0.54 (^−^1.34–0.26)	0.11 (^−^0.04–0.26)
12–17 hr	4.15 (0.08–8.21)^**^	^−^1.13 (^−^7.29–5.03)	0.26 (^−^0.47–0.99)	0.19 (0.05–0.32)^**^
18–23 hr	3.35 (^−^0.26–6.95)^*^	^−^2.37 (^−^6.93–2.18)	0.11 (^−^0.52–0.73)	0.09 (^−^0.03–0.22)
0–23 hr	5.79 (1.38–10.19)^#^	^−^2.72 (^−^9.27–3.83)	0.20 (^−^0.59–1.00)	0.16 (0.02–0.31)^**^
EC				
0–5 hr	1.97 (^−^1.79–5.73)	^−^4.67 (^−^10.00–0.67)^*^	0.52 (^−^0.16–1.21)	0.13 (0.00–0.26)^**^
6–11 hr	2.52 (^−^2.15–7.19)	^−^0.81 (^−^7.92–6.31)	^−^0.23 (^−^1.10–0.64)	0.10 (^−^0.06–0.26)
12–17 hr	1.94 (^−^1.58–5.46)	^−^1.15 (^−^6.56–4.25)	0.33 (^−^0.32–0.98)	0.16 (0.04–0.29)^**^
18–23 hr	1.88 (^−^1.54–5.30)	^−^2.92 (^−^7.31–1.47)	0.21 (^−^0.41–0.83)	0.10 (^−^0.01–0.22)^*^
0–23 hr	3.07 (^−^1.21–7.34)	^−^3.38 (^−^9.81–3.05)	0.42 (^−^0.40–1.23)	0.18 (0.03–0.32)^**^

All models included the categorical variables patient number and weekday, and in addition, the following variables: for QTc interval—trend loess (df = 6.0), temperature lag 3 loess (df = 6.2), relative humidity lag 1 polynomial (second order), and barometric pressure lag 1 linear; for T-wave complexity—trend loess (df = 2.3), temperature lag 3 polynomial (second order), relative humidity lag 3 loess (df = 6.7), and barometric pressure lag 0 polynomial (third order); for T-wave amplitude—trend polynomial (third order), temperature lag 2 linear, relative humidity lag 3 polynomial (third order), and barometric pressure lag 3 linear; for variability of T-wave complexity—trend loess (df = 3.4), temperature lag 3 loess (df = 4.6), relative humidity lag 2 polynomial (second order), and barometric pressure lag 0 loess (df = 4.1).

* *p* < 0.10 (borderline significant).

** *p* < 0.05 (significant).

# *p* < 0.01 (highly significant).
